# Anticipated effects of burosumab treatment on long-term clinical sequelae in XLH: expert perspectives

**DOI:** 10.3389/fendo.2023.1211426

**Published:** 2023-07-20

**Authors:** Lothar Seefried, Martin Biosse Duplan, Karine Briot, Michael T. Collins, Rachel Evans, Pablo Florenzano, Neil Hawkins, Muhammad Kassim Javaid, Robin Lachmann, Leanne M. Ward

**Affiliations:** ^1^ Orthopedic Department, University of Würzburg, Würzburg, Germany; ^2^ Service de Médecine Bucco-Dentaire, Hôpital Bretonneau, AP-HP, Paris, France; ^3^ UFR d’Odontologie, Université de Paris, Paris, France; ^4^ Institut Imagine, INSERM, Paris, France; ^5^ Department of Rheumatology, Hôpital Cochin, Université de Paris-Cité, Paris, France; ^6^ Skeletal Disorders and Mineral Homeostasis Section, National Institutes of Dental and Craniofacial Research, National Institutes of Health, Bethesda, MD, United States; ^7^ Health Economics, Visible Analytics, Oxford, United Kingdom; ^8^ Department of Endocrinology, Pontificia Universidad Católica de Chile, Santiago, Chile; ^9^ Department of Endocrinology, Centro Traslacional en Endocrinologia (CETREN-UC), Santiago, Chile; ^10^ Nuffield Department of Orthopaedics, Rheumatology and Musculoskeletal Sciences, University of Oxford, Oxford, United Kingdom; ^11^ Charles Dent Metabolic Unit, National Hospital for Neurology and Neurosurgery, London, United Kingdom; ^12^ Children’s Hospital of Eastern Ontario, University of Ottawa, Ottawa, ON, Canada

**Keywords:** burosumab, X-linked hypophosphatemia, fibroblast growth factor 23, phosphate metabolism, hypophosphatemia

## Abstract

X-linked hypophosphatemia (XLH) is a rare, progressive, genetic disease with multisystem impact that typically begins to manifest in early childhood. Two treatment options exist: oral phosphate in combination with active vitamin D (“conventional therapy”) and a fully human monoclonal anti-FGF23 antibody, burosumab. The clinical benefit of conventional therapy in adults is limited, and poor tolerance and complications are common. Burosumab was first approved as a treatment for XLH in 2018 and its disease-modifying benefits in clinical trials in children suggest burosumab treatment could also alter the disease course in adults. Without long-term clinical data on multiple XLH-related sequelae available, the results of an elicitation exercise are reported, in which eight global experts in XLH posited how long-term treatment with burosumab is anticipated to impact the life course of clinical sequelae in adults with XLH. Based on their clinical experiences, the available evidence and their disease understanding, the experts agreed that some long-term benefits of using burosumab are likely in adults with XLH even if they have a misaligned skeleton from childhood. Burosumab treatment is anticipated to reduce the incidence of fractures and halt the progression of clinical sequelae associated with conventional therapy. While the trajectories for established dental abscesses are not expected to improve with burosumab treatment, dental abscess development may be prevented. Starting treatment with burosumab in childhood to increase the likelihood of an aligned skeleton and continuation into and throughout adulthood to maintain euphosphatemia may optimize patient outcomes, although future real-world investigation is required to support this hypothesis.

## 1 Introduction

X-linked hypophosphatemia (XLH) is a complex, rare, progressive, genetic disease characterized by loss-of-function mutations in the *PHEX* (phosphate-regulating gene with homologies to endopeptidases on the X chromosome) gene (1–3), leading to increased serum/plasma levels of fibroblast growth factor 23 (FGF23) (3). Elevated FGF23 impairs phosphate reabsorption by reducing expression of the NaP IIa/c co-transporters in the renal proximal tubules, leading to increased phosphate excretion and chronic hypophosphatemia (4–6). Increased FGF23 levels are also associated with lower levels of active vitamin D (1,25(OH)_2_D) due to decreased synthesis and increased catabolism leading to reduced gastrointestinal phosphate absorption, and further overall compromised mineral homeostasis and chronic hypophosphatemia (3, 4).

XLH typically manifests in early childhood, resulting in rickets, skeletal deformities, short stature (7–9), and dental abscesses in many children (10, 11). Chronic hypophosphatemia and osteomalacia combined with the irreversible skeletal deformities acquired in childhood are linked to progressive clinical sequelae in adulthood, including osteoarthritis, enthesopathies, osteomalacic fractures, and spinal stenosis (10, 12), with the prevalence and severity of many increasing with age (8, 13–15). Given the multisystem impact of XLH, it is unsurprising that adults with XLH suffer from considerable chronic pain, impaired mobility, loss of physical function and hearing, and decreased health-related quality of life (16–20).

Conventional therapy consists of supplementation with oral phosphate in combination with active vitamin D (21). This therapy has been investigated much more extensively in children than in adults (22–25). Conventional treatment can improve symptomatic, histomorphometric, and growth outcomes (22, 25). However, even with coherent therapy, 25–40% of patients experience linear growth failure (23). Conventional treatment does not restore phosphate homeostasis (26); it results in only transient improvement in serum phosphate and active vitamin D levels. Concurrently, treatment is often poorly tolerated, and complications can result, e.g., secondary and tertiary (hypercalcemic) hyperparathyroidism and nephrocalcinosis (10, 21, 27–29).

Burosumab, first approved as a treatment for XLH in 2018 in the United States and for children in Europe, then as of 2020 also for the treatment of adults with XLH in Europe, is a fully human, immunoglobulin G1 monoclonal antibody that binds to and inhibits excess FGF23 activity, enabling restoration of serum phosphate levels (30–32). In a randomized, double-blind, Phase III trial, adults (18−65 years) with XLH and baseline serum phosphate concentrations below the lower limit of normal (LLN; <2.5 mg/dL) received 1.0 mg/kg burosumab every 4 weeks for 24 weeks or a placebo. By study end, 94.1% of patients receiving burosumab achieved a mean serum phosphate concentration with the midpoint of the dose interval above the LLN (subsequently termed ‘serum phosphate normalization’) compared to 7.6% receiving placebo. Significantly more patients randomized to burosumab than placebo experienced full healing of fractures active at baseline by study end (43.1% vs 7.7%; p<0.001) (30, 31).

Burosumab was also associated with improvements in the Brief Pain Inventory (BPI) (33) for worst pain scores and Western Ontario and the McMaster Universities Osteoarthritis Index (WOMAC) physical function and stiffness subscales (34); the latter reached statistical significance (30). In the open-label extension studies with all patients receiving burosumab over 96 weeks in total, treatment was associated with persistent improvements regarding pain, stiffness, and physical function and further healing of pseudofractures (31). Burosumab has also been associated with significant improvements in histomorphometric parameters of osteomalacia in adults by 48 weeks compared to baseline (35).

Persistent improvements of clinical outcomes with burosumab over 96 weeks suggest that continuous treatment in adults may change the life course of the disease, preventing future events or slowing progression of XLH-related sequelae. However, long-term clinical data substantiating this change in life course are not yet available. Furthermore, XLH may impact more than the specified pain, stiffness, and fracture healing outcomes within clinical trials. Thus, opinions were sought from eight experts in XLH to posit how long-term treatment with burosumab is anticipated to impact the life course of clinical sequelae in adults with XLH.

## 2 Materials and methods

### 2.1 Questionnaire

A questionnaire was designed to explore insights into putative causal links between XLH pathophysiology and clinical implications, in accordance with appropriate Health Technology Assessment (HTA) guidelines (36–39). Seven clinical experts considered numerous clinical sequelae including fractures (31), enthesopathy, spinal stenosis, tinnitus/hearing loss, osteoarthritis, osteophytes, and dental abscesses. In addition, the course of three clinical consequences understood to be associated with conventional therapy – nephrocalcinosis, kidney stones, and hyperparathyroidism – were considered.

**Figure 1:**
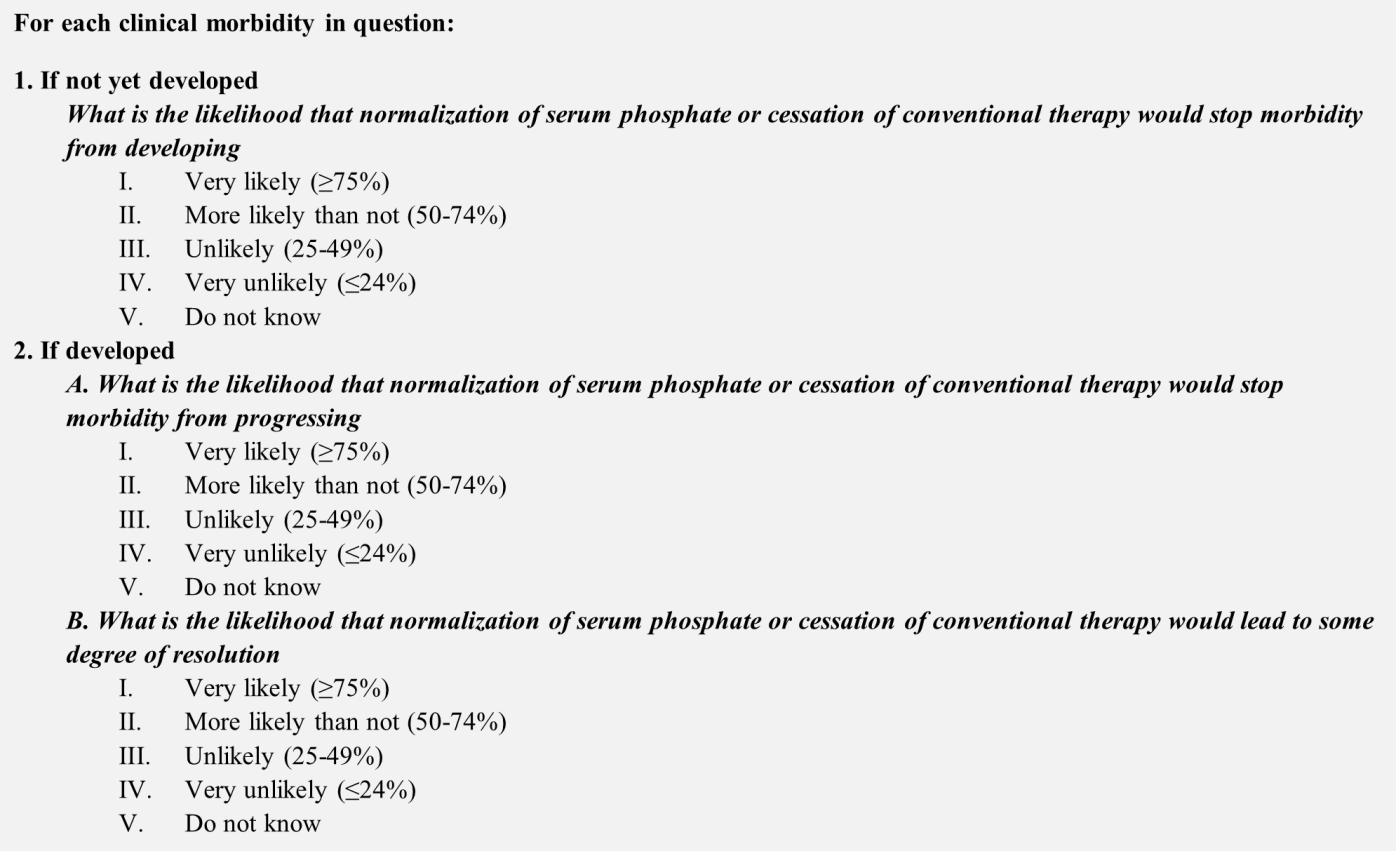
Format of questionnaire.

**Figure 2:**
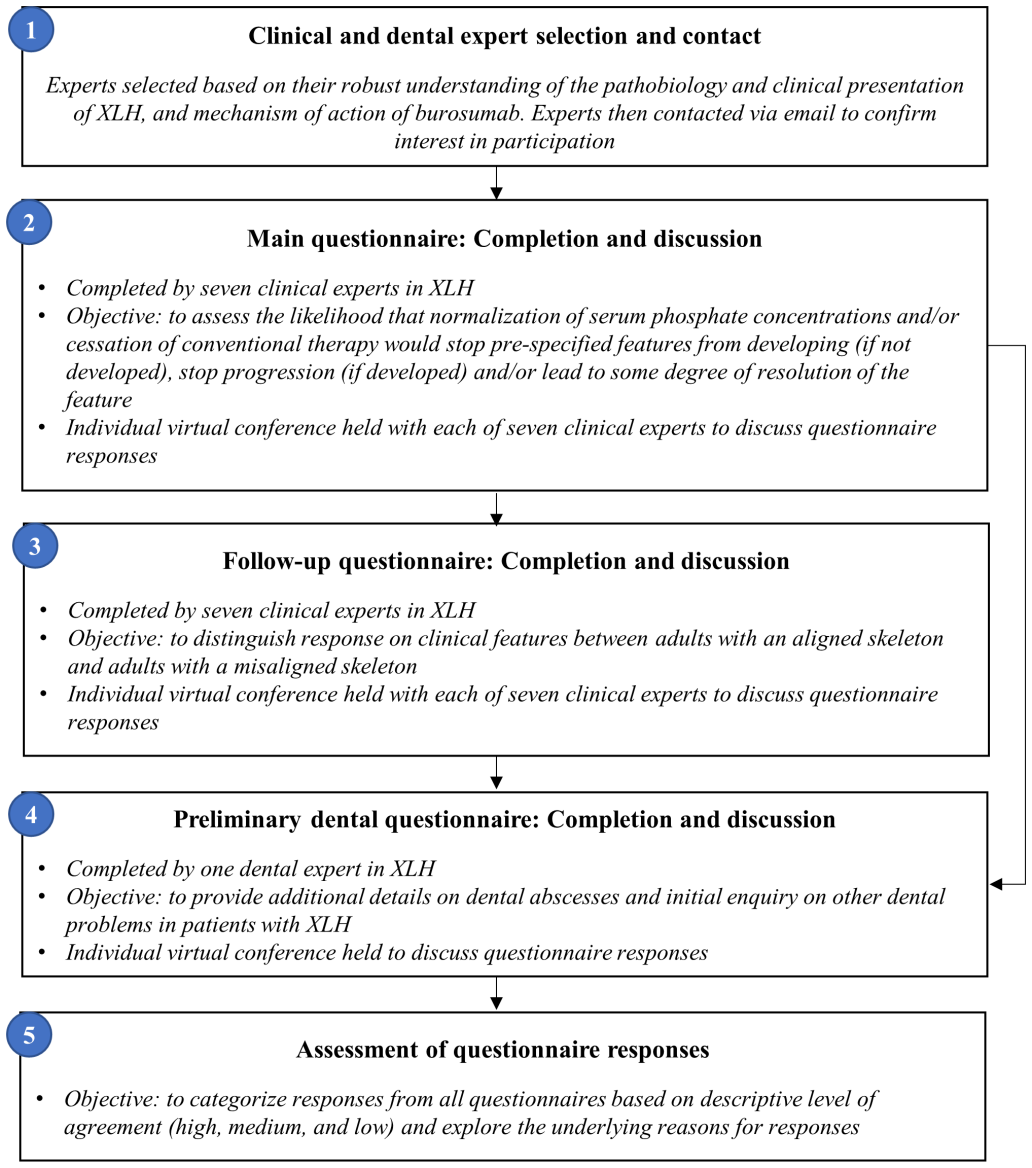
Flowchart of expert elicitation process.

Experts were asked to assess the likelihood that normalization of serum phosphate and/or cessation of conventional therapy (a proxy for burosumab treatment), would stop each clinical sequela from developing if not already developed (hereafter referred to as ‘prevent development’), and/or stop each clinical sequela from progressing (hereafter referred to as ‘halt progression’) and/or lead to resolution of developed clinical sequelae (hereafter referred to as ‘reverse’) (**Figure 1**). Experts provided their answers by completing an individual questionnaire using a five-point rating scale: i) Very likely (>75%); ii) More likely than not (50−74%); iii) Unlikely (25−49%); iv) Very unlikely (<24%); or v) Do not know (**Figure 2**).

Following completion of the main questionnaire, a musculoskeletal follow-up questionnaire was issued, distinguishing between adults with an aligned (i.e., without skeletal deformity) or misaligned skeleton (**Figure 2**), to differentially evaluate clinical manifestations thought to be affected by a misaligned skeleton (fractures, enthesopathy, spinal stenosis, osteoarthritis, osteophytes). Consistency between responses was descriptively assessed and collated within the results. In addition, a preliminary questionnaire was sent to a dental expert for additional insight into dental sequelae.

Due to the ongoing COVID-19 pandemic, all interviews were conducted virtually. Following the elicitation exercises, an online discussion platform was set up by ApotheCom (with only them having ability to directly pose questions to and discuss with experts). This platform intended to emulate in-person dialog between the clinical experts, and gather clinical experience and scientific literature to support their responses on the utility of burosumab across clinical sequelae. The sponsor was not involved in these dialogs with the experts, nor were they involved in the subsequent drafting of the manuscript.

### 2.2 Clinical and dental expert selection

Given that burosumab is the first approved treatment for XLH in children and adults and this is a complex, rare disease affecting 1 in 20,000 people, the sponsor has previously collaborated with multiple clinicians and dentists who are dedicated to patient care in the field. However, the structured elicitation exercise forming the basis of this manuscript was an unrelated initiative to those previous collaborations; experts were selected by the sponsor to participate in the elicitation exercise based on their robust understanding of the pathobiology and clinical presentation of XLH, and mechanism of action of burosumab. In the absence of known pathophysiology, experts were advised to answer the questions based on clinical experience. Expert knowledge was determined through a comprehensive publication history relevant to phosphate regulation and physiological outcomes associated with hypophosphatemia, and/or clinical experience in the management of patients with XLH. A broad opinion base was achieved by seeking heterogeneity in expert specialties and their geographical regions of clinical practice.

A total of eight identified experts were contacted via email by the sponsor and all agreed to participate; seven clinical experts and one dental expert. From that point on, a third-party company (Visible Analytics Ltd) was contracted to conduct the questionnaires independently without input from the sponsor. Between 19 June 2020 and 5 November 2020, the questionnaires were sent to each participant followed by an individual videoconference hosted by Visible Analytics Ltd to further discuss the questionnaire responses. During the exercise, the clinical experts’ responses were not known to each other, thereby ensuring independent answers.

## 3 Results

Seven clinical experts, with input from one dental expert, from the UK, USA, Chile, France, Canada, and Germany completed the first and second musculoskeletal questionnaires. The dental expert from France completed the preliminary dental questionnaire. Clinician responses were categorized based on the descriptive level of agreement; high (**Supplementary Figure 1**), medium (**Supplementary Figure 2**), and low (**Supplementary Figure 3**).

### 3.1 High level of agreement

Treatment with burosumab was considered very likely to stop the development of all future fractures regardless of fracture history. Burosumab was also very likely to specifically prevent future lower limb/hip fractures, halt progression of existing lower limb fractures, and reverse existing fractures. At least some resolution was expected to be very or more likely than not to occur in patients with both early and well-established fracture histories. Burosumab was expected to more likely than not or very likely prevent future fractures in adults with a misaligned skeleton; prevention would be even more likely in adults with an aligned skeleton.

In adults without osteoarthritis and osteophytes, treatment with burosumab was thought very likely/more likely than not by most clinicians to prevent development, and development of each complication was rated unlikely/very unlikely by only one clinical expert. It was thought unlikely to prevent development in adults with a misaligned skeleton, and very likely in adults with an aligned skeleton. If adults had early or well-established osteoarthritis or osteophytes, treatment with burosumab was considered unlikely to halt further progression and very unlikely to reverse osteoarthritis or osteophytes.

If nephrocalcinosis, kidney stones, and secondary hyperparathyroidism had not yet developed, treatment with burosumab was thought very likely to prevent future development. If adults had early development of these conditions, most of the clinicians considered that treating with burosumab would more likely than not halt further progression and it is very likely/more than likely to reverse the side effects. If adults had well-established nephrocalcinosis or kidney stones, treatment with burosumab was rated as being likely to halt further progression but very unlikely to reverse these complications. However, most clinicians considered treatment with burosumab as unlikely to halt progression or reverse tertiary hyperparathyroidism.

### 3.2 Medium level of agreement

Treatment with burosumab was considered very likely/more likely than not to prevent development of enthesopathies, even in adults with a misaligned skeleton. If adults had early development of enthesopathy, burosumab treatment was predominantly thought to be more likely than not to halt further progression, but opinion was divided as to whether such treatment would reverse enthesopathies. In patients with well-established enthesopathy, burosumab was considered unlikely/very unlikely to halt further progression or reverse enthesopathies. The likelihood of halting further progression was judged to be greater in adults with an aligned vs misaligned skeleton.

Similarly, if spinal stenosis had not yet developed, treatment with burosumab was considered more likely than not or very likely to prevent development, with prevention ‘very likely’ if an adult has an aligned skeleton. If adults had early development of spinal stenosis, treatment with burosumab was rated as more likely than not to halt further progression by most clinicians, even with a misaligned skeleton, and more likely than not to halt progression if there is an aligned skeleton. In contrast, burosumab was considered unlikely to prevent progression and very unlikely to reverse well-established spinal stenosis. Reversal of spinal stenosis was considered unlikely. If adults had well-established spinal stenosis, treatment with burosumab was considered unlikely to prevent progression and very unlikely to reverse stenosis.

### 3.3 Low level of agreement

If tinnitus/hearing loss had not yet developed, most of the clinicians thought burosumab treatment would be very likely/more likely than not to prevent development. If adults had early development of tinnitus/hearing loss, treatment with burosumab was considered more likely than not to halt further progression by most clinicians, but unlikely to cause resolution. In adults with well-established tinnitus/hearing loss, four of the clinical experts thought that burosumab treatment was likely to halt progression but two thought it unlikely. All clinicians thought burosumab unlikely/very unlikely to reverse tinnitus.

### 3.4 Dental abscesses

In adults without developed dental abscesses, burosumab treatment was thought by most to be very likely/more likely than not to prevent development (**Supplementary Figure 4**). If adults had early development of dental abscesses, some clinicians considered that such treatment would be likely to halt further progression, whereas others considered this to be unlikely; there was consensus that burosumab treatment would be unlikely to lead to reversal of dental abscesses. If adults had well-established dental abscesses, it was agreed that burosumab treatment would be unlikely to halt progression or cause any reversal.

## 4 Discussion

Given that burosumab was first approved for XLH in 2018, long-term data on the use within adults is not yet available. Therefore, this publication explores the anticipated effects that burosumab may have on clinical outcomes in XLH through expert opinion and a robust methodology linked to the available science. The aim of this clinical expert elicitation exercise was not to reach consensus. Differences in opinions were expected, and reflect uncertainties arising from the differences in interpreting the evidence (where available) and extrapolating from clinical experiences, and complexity of the disease.

Experts broadly agreed on the potential benefits of burosumab on numerous long-term clinical and radiographic sequelae that manifest in adults with XLH. Overall, treatment with burosumab was expected to prevent the development of numerous morbidities, specifically fractures, enthesopathy, spinal stenosis and tinnitus/hearing loss and potentially halt the progression of fractures, and early stages of enthesopathy, spinal stenosis and tinnitus/hearing loss while burosumab was considered less likely to halt further progression or to reverse already established sequelae, particularly osteoarthritis, osteophytes, but also advances, well-established enthesopathy, spinal stenosis or hearing loss/tinnitus. There was considerable agreement that switching treatment to burosumab would not reverse well-established manifestations associated with conventional therapy, such as tertiary hyperparathyroidism, but it would prevent development of and could potentially halt further progression of nephrocalcinosis and kidney stones. In fact, switching from conventional to burosumab therapy may prevent nephrocalcinosis in children (40).

Given that elevated FGF23 in XLH is the cause of hypophosphatemia, with the latter being a major driver of osteomalacia and fractures in adults (10), neutralization of FGF23 with burosumab, and restoring serum phosphate levels, was anticipated to potentially reduce the risk of future fractures. Restoration of phosphate homeostasis should allow the skeleton to undergo proper mineralization, and thereby withstand the typical mechanical loads of day-to-day life. In support, the pivotal adult trial of burosumab demonstrated significantly higher percentages of fracture healing with burosumab compared to placebo (16.8-fold higher at 24 weeks) (30, 31). Based on this strong scientific rationale, all clinicians indicated that treatment with burosumab would likely/very likely inhibit the development of future fractures at all skeletal sites, even in adults with a misaligned skeleton.

The preventive effect of burosumab on the development of enthesopathies and/or spinal stenosis was considered most likely if these were not present, yet, or in an early stage of development. The outlook is most positive for adults with an aligned skeleton, thus underscoring the critical role of optimal treatment in children and, in preventing long bone deformity as they grow. The greater diversity in clinician responses on these questions compared to the unanimity regarding the effect on fractures probably alludes to less certainty in the scientific rationale. Enthesopathies may arise as a biomechanical adaption to abnormal mechanical stresses, as in osteomalacia (41), wherein the ability of the enthesis to accommodate and dissipate stress at the tendon-bone interface is compromised (41, 42). This can lead to pain and limitations in range of motion (43). In light of burosumab restoring phosphate levels and osteomalacia-related histomorphometric measures (35), this might prospectively mitigate the risk of progressive deformity when treatment is started early.

Spine abnormalities in XLH, may potentially be due to abnormal strains on paraspinal ligaments causing mineralization of entheses (43, 44), therefore, improved skeletal mineralization and balanced mineral metabolism with burosumab might decrease spine enthesopathy. Since mineralization of the paraspinal ligaments is not an inherent aspect of other disease groups with spinal deformity, systemic disturbance of mineral homeostasis or even a direct effect of excess FGF23 on tissue mineralization might contribute to calcification of the paraspinal ligaments in XLH, and hence respond to burosumab. This would also apply if calcification of soft tissues (e.g., ligaments) was directly related to excess FGF23 and its effect on target tissues, otherwise, if spinal stenosis is primarily related to osteoarthritis in the spine, it would likely not be amenable to treatment with burosumab.

Hearing loss and tinnitus are other known clinical consequences of XLH (19, 20, 43, 45), which can reduce health-related quality of life (46). It is hypothesized that hypophosphatemia and osteomalacia associated with increased FGF23 in XLH can lead to temporal bone malformation. Animal models suggest that FGF23 is essential for normal development of the middle ear and functioning of the middle and inner ear, and that FGF23 deficiency may predispose to otitis media (3, 47). Although recent findings highlight the importance of ossicular mineralization for hearing conduction, the complex pathophysiology behind hearing loss in XLH is still elusive (3, 48). Hearing loss/tinnitus in XLH is not completely understood, yet burosumab was considered more likely than not to help stop development or progression, respectively, but suspected unlikely to resolve already established hearing loss.

Osteoarthritis is likely a matter of XLH-related alterations of joint and cartilage development (41, 44, 49, 50), and a long-term consequence of weight bearing on misaligned hips, knees, and ankles in adults (19, 50, 51). In animal models, restoration of a mineralizing zone of articular chondrocytes (and markers of mineralized chondrocytes) by phosphate replacement therapy suggest that serum phosphate management may be important in both early intervention and long-term management of osteoarthritis-related complications of XLH (41). Additionally, FGF23 may have a direct role in cartilage development (52). Thus starting treatment with burosumab only in adulthood will presumably not halt or reverse already developed osteoarthritis; but it may limit consequences of FGF23 excess on cartilage and prevent joint degeneration in case treatment is started before osteoarthritis is at an advanced stage.

Finally, dental abscesses occur at a high rate in children and adults with XLH (19, 53) (51% and 82%, respectively) (19). One of the main drivers of dental abscesses in XLH appears to be defective dentin mineralization (54, 55). Parameters influencing the effectiveness of XLH therapies in children, such as the age at treatment onset and compliance, may also impact the occurrence of dental abscesses in adults. As conventional therapy that is continued during adulthood may reduce dental abscesses (56), it is possible that burosumab treatment, which restores euphosphatemia, also decreases the occurrence of dental abscesses (57). Experts believed that dental abscesses may be prevented if not yet developed when burosumab treatment is given. In those with already developed dental abscesses, treatment with burosumab was thought unlikely to alter the risk of new dental abscesses.

Clinical sequelae considered associated with conventional therapy for XLH include nephrocalcinosis, kidney stones, and secondary hyperparathyroidism (3, 24, 28, 29, 43). Thus cessation of conventional therapy in favor of burosumab, was considered likely to slow progression and possibly lead to some resolution of these manifestations unless already well established. Obtaining longitudinal real-world data from observational studies and established registries (NCT03193476, NCT03651505) will be essential to better understand long-term effects of treatments for XLH (58).

The long-term outcomes with burosumab hypothesized in this manuscript reflect a synthesis of the experience and disease perception of the contributing experts. However, their opinions may not reflect others in their respective fields. Furthermore, each participating clinician had their own areas of expertise which may have influenced their responses. While a structured questionnaire and online discussion platform were used to enable individual-level sharing and discussions, in-person discussions (which were prohibited by the COVID-19 global circumstances during the course of this exercise) may have been beneficial. Overall, this work provides impetus for future studies that seek to validate the hypotheses and ideas put forward by the clinical experts in this elicitation exercise.

## Data availability statement

The original contributions presented in the study are included in the article/**Supplementary Material**. Further inquiries can be directed to the corresponding author.

## Ethics statement

Ethics approval and informed consent were not required due to the nature of this study, which involved the analysis of expert perspectives and published literature only.

## Author contributions

RE and NH: Designing of methodology and implementation of the elicitation exercise. LS, MD, KB, MC, PF, MJ, RL, LW, RE and NH: Writing - Original draft preparation, reviewing and editing of subsequent drafts based on substantive expertise in the topic area. All authors contributed to the article and approved the submitted version.
