# Supplementary material for: Anticipated effects of burosumab treatment on long-term clinical sequelae in XLH: expert perspectives
Source: Front Endocrinol (Lausanne). 2023 Jul 20;14:1211426. doi: 10.3389/fendo.2023.1211426 (PMC10400326; doi:10.3389/fendo.2023.1211426)
Supplement: Supplementary file 1 [file DataSheet_1.zip › Supplementary figure 2_V1 05.10.22.docx]

Supplementary Figure 2. What is the likelihood that normalization of serum phosphate or cessation of conventional therapy would lead to some degree of resolution? (Medium level of expert agreement). Each triangle indicates one expert response.

| **Enthesopathy** | | | | | |
| --- | --- | --- | --- | --- | --- |
|  | **Very likely (>75%)** | **More likely than not (50−74%)** | **Unlikely (25−49%)** | **Very unlikely (<24%)** | **Do not know** |
| **Not yet developed**  Prevent development | ▲▲▲▲ | ▲▲▲ |  |  |  |
| **Early development**  Halt further progression | ▲ | ▲▲▲▲▲ | ▲ |  |  |
| **Early development**  Reverse |  | ▲▲▲ | ▲▲▲ | ▲ |  |
| **Well-established**  Halt further progression | ▲ | ▲▲ | ▲▲▲ | ▲ |  |
| **Well established**  Reverse | ▲ | ▲ | ▲▲ | ▲▲▲ |  |

| **Enthesopathy** | | | | | |
| --- | --- | --- | --- | --- | --- |
|  | **Very likely (>75%)** | **More likely than not (50−74%)** | **Unlikely (25−49%)** | **Very unlikely (<24%)** | **Do not know** |
| *Misaligned skeleton* | | | | | |
| **Not yet developed**  Prevent development | ▲▲ | ▲▲▲▲ | ▲ |  |  |
| **Developed**  Halt further progression |  | ▲▲▲▲▲ | ▲▲ |  |  |
| *Aligned skeleton* | | | | | |
| **Not yet developed**  Prevent development | ▲▲▲ | ▲▲▲▲ |  |  |  |
| **Developed**  Halt further progression |  | ▲▲▲▲▲▲▲ |  |  |  |

| **Spinal stenosis** | | | | | |
| --- | --- | --- | --- | --- | --- |
|  | **Very likely (>75%)** | **More likely than not (50−74%)** | **Unlikely (25−49%)** | **Very unlikely (<24%)** | **Do not know** |
| **Not yet developed**  Prevent development | ▲▲ | ▲▲▲ | ▲ |  | ▲ |
| **Early development**  Halt further progression |  | ▲▲▲▲▲ | ▲▲ |  |  |
| **Early development**  Reverse |  |  | ▲▲▲▲ | ▲▲▲ |  |
| **Well-established**  Halt further progression |  | ▲▲▲ | ▲▲▲▲ |  |  |
| **Well established**  Reverse |  |  | ▲ | ▲▲▲▲▲▲ |  |

| **Spinal stenosis** | | | | | |
| --- | --- | --- | --- | --- | --- |
|  | **Very likely (>75%)** | **More likely than not (50−74%)** | **Unlikely (25−49%)** | **Very unlikely (<24%)** | **Do not know** |
| *Misaligned skeleton* | | | | | |
| **Not yet developed**  Prevent development | ▲ | ▲▲▲▲ | ▲▲ |  |  |
| **Developed**  Halt further progression | ▲ | ▲▲ | ▲▲▲ | ▲ |  |
| *Aligned skeleton* | | | | | |
| **Not yet developed**  Prevent development | ▲▲▲▲ | ▲▲ | ▲ |  |  |
| **Developed**  Halt further progression |  | ▲▲▲▲ | ▲▲▲ |  |  |
