# Supplementary material for: Anticipated effects of burosumab treatment on long-term clinical sequelae in XLH: expert perspectives
Source: Front Endocrinol (Lausanne). 2023 Jul 20;14:1211426. doi: 10.3389/fendo.2023.1211426 (PMC10400326; doi:10.3389/fendo.2023.1211426)
Supplement: Supplementary file 1 [file DataSheet_1.zip › Supplementary figure 3_V1 05.10.22.docx]

Supplementary Figure 3. What is the likelihood that normalization of serum phosphate or cessation of conventional therapy would lead to some degree of resolution? (Low level of expert agreement). Each triangle indicates one expert response.

| **Tinnitus/hearing loss** | | | | | |
| --- | --- | --- | --- | --- | --- |
|  | **Very likely (>75%)** | **More likely than not (50−74%)** | **Unlikely (25−49%)** | **Very unlikely (<24%)** | **Do not know** |
| **Not yet developed**  Prevent development | ▲▲▲▲ | ▲ | ▲ |  | ▲ |
| **Early development**  Halt further progression | ▲ | ▲▲▲▲ | ▲ |  | ▲ |
| **Early development**  Reverse |  |  | ▲▲▲▲ | ▲▲ | ▲ |
| **Well-established**  Halt further progression | ▲ | ▲▲▲ | ▲▲ |  | ▲ |
| **Well established**  Reverse |  |  | ▲▲▲ | ▲▲▲ | ▲ |
